# Deciphering the performance of different surface models for corneal topography

**DOI:** 10.1111/opo.13539

**Published:** 2025-06-19

**Authors:** Achim Langenbucher, Nóra Szentmáry, Alan Cayless, Peter Hoffmann, Jascha Wendelstein

**Affiliations:** ^1^ Department of Experimental Ophthalmology Saarland University Homburg/Saar Germany; ^2^ Dr. Rolf M. Schwiete Centre for Limbal Stem Cell and Aniridia Research Saarland University Homburg/Saar Germany; ^3^ Department of Ophthalmology Semmelweis‐University Budapest Hungary; ^4^ School of Physical Sciences The Open University Milton Keynes UK; ^5^ Augen‐ Und Laserklinik Castrop‐Rauxel Castrop‐Rauxel Germany; ^6^ Department of Ophthalmology LMU Klinikum Munich Germany

**Keywords:** corneal surface fitting, Gaussian process surface, model surfaces, raytracing, Zernike surface

## Abstract

**Purpose:**

To study the performance of different corneal surface models to be used for ray tracing. Models based on geometric surfaces and polynomial fits were compared and the differences discussed.

**Methods:**

For this simulation study, five characteristic generic surface configurations were generated: (A) perfect biconic, (B) decentred biconic with white noise, (C) biconic with paracentral hollow simulating the situation after myopic LASIK, (D) biconic with random dot irregularities and (E) rotationally symmetric conic with mid‐peripheral bump simulating the situation of corneal ectasia. A floating best fit sphere (BFS), conic (BFC), biconic (BFBC), fringe Zernike on top of a BFS (BFSZ), fringe Zernike (BFZ) and Gaussian process surface model (BFGP) were fitted and the root‐mean‐squared fit error was analysed.

**Results:**

Surfaces A and B were well described by BFBC, BFSZ, BFZ and BFGP, but not by BFS and BFC. Surface C was not well represented by BFS, BFC and BFBC, but reasonably with BFSZ and BFZ and quite well with BFGP. Surfaces D and E were poorly represented, especially with BFS, BFC and BFBC, but also with BFSZ and BFZ and quite well with BFGP. There was no systematic difference between the two Zernike representations BFSZ and BFZ, even for surface B.

**Conclusions:**

Representing corneal point cloud data with a closed surface model plays a key role in ray tracing. Simple surface models such as BFS, BFC or BFBC are easy to handle but do not fully represent clinical situations with local irregularities after corneal refractive surgery or with ectasia.

**Key points**

- Ray tracing for corneal modelling requires continuous differentiable surfaces, but topographers and tomographers measure the corneal surface only at discrete points.
- Six methods of fitting continuous surfaces to discrete corneal data were evaluated using simulated profiles representing typical corneal situations.
- Zernike models were powerful, but the Gaussian process models had the best all‐round performance.

## BACKGROUND

Ray tracing is a valuable tool for evaluating the imaging performance of optical systems.^1^ A bundle of rays is projected onto the first refractive surface and sequentially traced through the entire system to derive ray scatter at the image plane. Relevant parameters such as the point spread function or the modulation transfer function can be extracted from the wavefront characteristics in the pupillary plane, and the transference of objects (in object space) to the corresponding image (in image space) can be estimated either by convolution of the object with the point spread function or by multiplication of the Fourier transformed object and the modulation transfer function.^1^, ^2^

In raytracing applications, the 3D coordinates of the ray‐surface intersection and the corresponding normal surface have to be calculated for each incident ray. This implies that a closed and continuous definition of the surface is required.^1^, ^2^ However, corneal topographers (or tomographers) only measure the corneal front and back surfaces at complete or incomplete^3^ discrete sampling points, meaning that the surface is not fully described for all possible ray intersection points. All topographers and tomographers on the market export surface curvature or height data. These are generally provided either in a Cartesian grid with lateral X and Y coordinates, surface curvature or height in the Z direction, or in a cylindrical grid organised in meridians with the distance from the axis, meridional angle and surface curvature or height in the Z direction.^1^, ^4^

There is no unique definition of a closed refractive surface based on these grid points. If the measurement data were free from noise, then one could apply any interpolation technique between the grid points, including linear De Launey triangulation^5^ or bicubic splines.^2^ However, interpolation of the grid data is not appropriate where noise is present in the data, and therefore, one has to apply approximation techniques, meaning that the fitted model surface would not necessarily pass through all of the grid points. There are various options for model surfaces,^6^, ^7^ including (floating) best fit spheres (BFS), second‐order conic surfaces (BFC) such as ellipsoids, paraboloids or hyperboloids,^4^, ^8^ biconic surfaces (BFB), wavelet surfaces,^9^ polynomial surfaces such as Zernike models (BFZ)^10^, ^11^, ^12^ or Gaussian process surfaces (BFGP).^7^, ^13^, ^14^, ^15^, ^16^, ^17^, ^18^

These surfaces are characterised by parameters specific to each model. In the case of the generic surfaces, these are the radius R and the X/Y/Z coordinates of the apex (BFS), R and asphericity (Q) (BFC) or the radii (R1 and R2) and asphericity (Q1 and Q2) in the flat and steep meridians, together with the orientation of the flat meridian (A1) (biconic – BFBC). For the Zernike and Gaussian fits, these are the Zernike coefficients (BFZ) or weights and biases (BFGP). Since Zernike surfaces are defined in the unit circle only, some normalisation is required.^10^, ^11^ Additionally, since Zernike surfaces are not designed to cover lateral displacement, in many cases they are used on top of a simple model surface such as a floating BFS.

All of these model surfaces used for characterising the front and back surface of the cornea have advantages and drawbacks in terms of the complexity of the surface fit or ray tracing strategy,^6^ or the degree of customisation to any surface data set from a topographer or tomographer.^12^ As an example, fitting a BFS to topographic data and calculating the ray surface intersection and surface normal with a BFS is very simple and straightforward.^8^ By contrast, a surface fit with a BFBC (with five degrees of freedom) or a BFGP is much more flexible but requires nonlinear iterative optimisation techniques.

The purpose of the present study was to evaluate several different corneal surface models to be used for ray tracing. The benefits and drawbacks of the different models are discussed in terms of fit error and tolerance to local irregularity or decentration, using selected generic surface data as examples.

## METHODS

**FIGURE 1:**
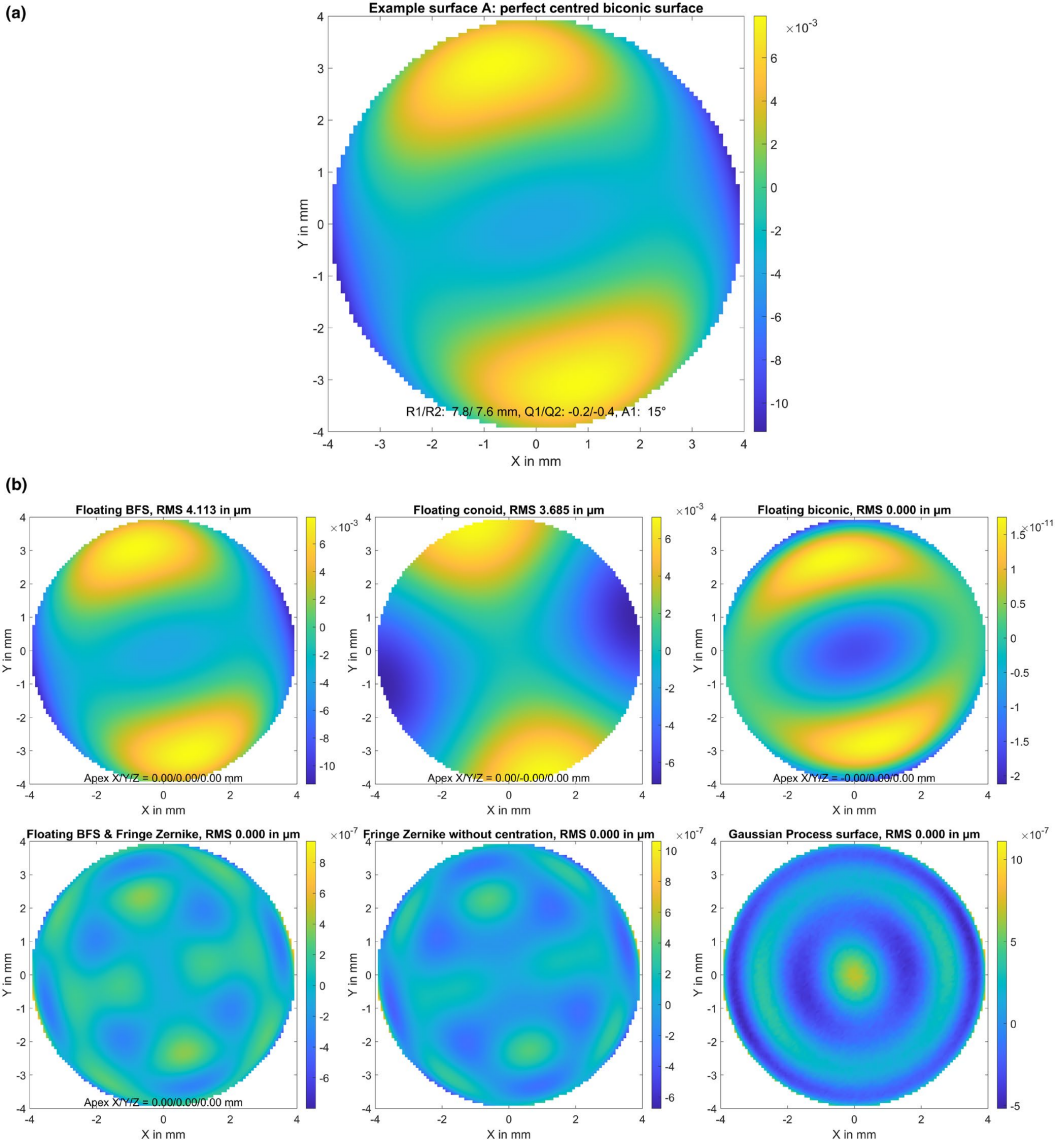
(a) Elevation maps of the generic surface A (perfect centred biconic surface, the flat meridian is oriented at 15 degrees) in terms of height difference from a best fit floating sphere. The surface is defined on a Cartesian grid within the central 8 mm zone with a lateral sampling of 100 points in both X and Y (in total 7668 data points). Dimensions for X, Y and Z data are in mm. (b) Fit error (FE) with a best fit floating spherical surface model (BFS, upper left graph), a best fit floating conic surface model (BFC, upper middle graph), a best fit floating biconic surface model (BFBC, upper right graph), a best fit floating spherical surface (as defined with BFS) with a fringe Zernike surface of radial degree N_Z_ = 12 on top (BFSZ, lower left graph), a fringe Zernike surface model of radial degree N_Z_ = 12 (BFZ, lower middle graph) and a custom Gaussian process surface model (BFGP, lower right graph) for surface A. The root‐mean‐squared fit error is shown in a millimetre scale, and the root‐mean‐squared fit error (RMS) is provided in the title of the graph. Note that the colour maps are normalised in each case to the range of values in each plot.

**FIGURE 2:**
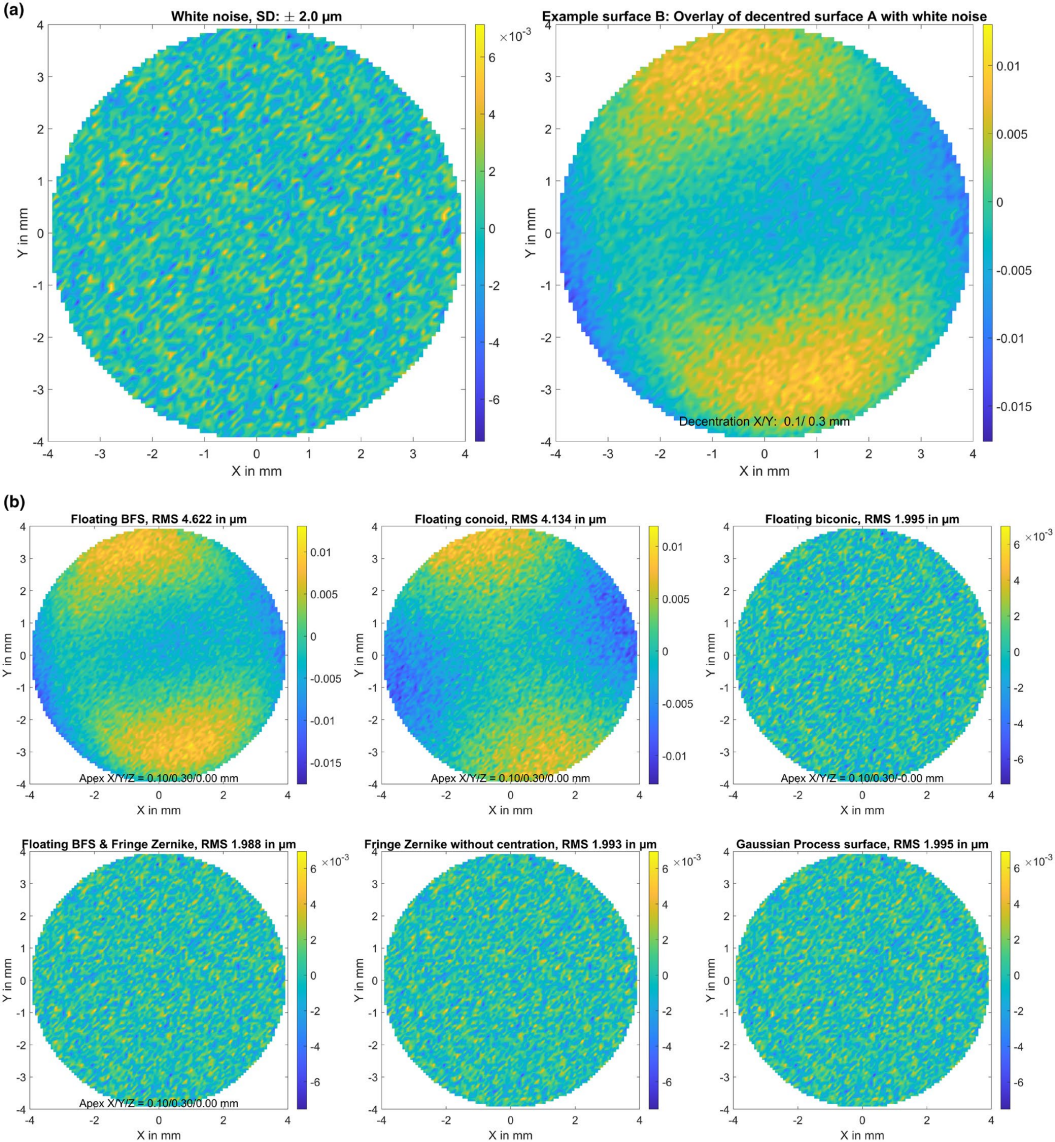
(a) Elevation maps of the generic surface B (the biconic surface from Figure 1a but with decentration of 0.1 mm in X and 0.2 mm in Y and a superposition of white random noise (left graph) with a standard deviation of 2 μm) in terms of height difference from a best fit floating sphere. The surface is defined on a Cartesian grid within the central 8 mm zone with a lateral sampling of 100 points in both X and Y (in total 7668 data points). Dimensions for X, Y and Z data are in mm. (b) Fit error (FE) with a best fit floating spherical surface model (BFS, upper left graph), a best fit floating conic surface model (BFC, upper middle graph), a best fit floating biconic surface model (BFBC, upper right graph), a best fit floating spherical surface (as defined with BFS) with a fringe Zernike surface of radial degree N_Z_ = 12 on top (BFSZ, lower left graph), a fringe Zernike surface model of radial degree N_Z_ = 12 (BFZ, lower middle graph) and a custom Gaussian process surface model (BFGP, lower right graph) for surface B. The root‐mean‐squared fit error is shown in a millimetre scale and the root‐mean‐squared fit error (RMS) is provided in the title of the graph. Note that the colour maps are normalised in each case to the range of values in each plot.

**FIGURE 3:**
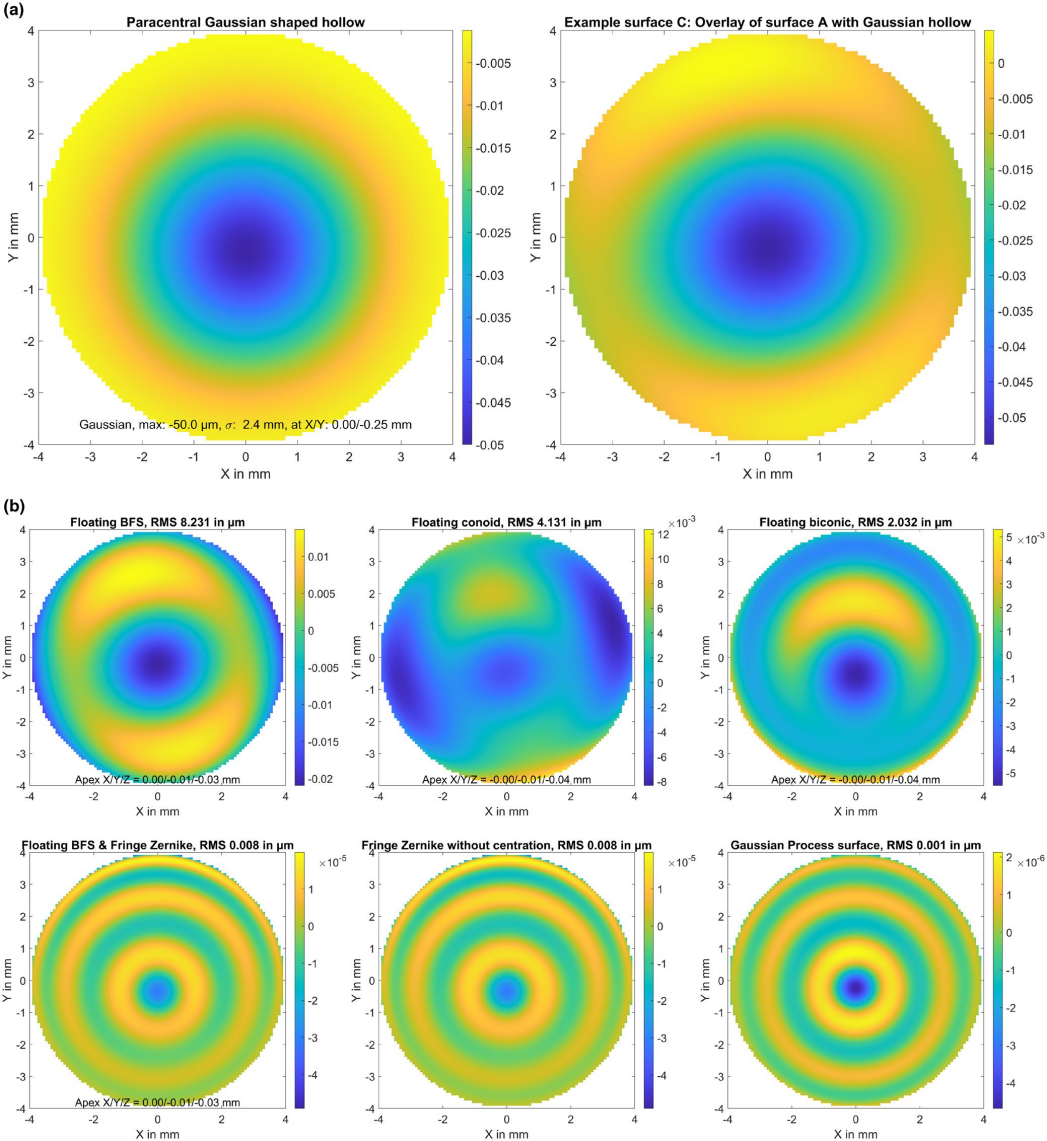
(a) Elevation maps of the generic surface C in terms of height difference from a best fit floating sphere. Surface C refers to the biconic surface from Figure 1a with a superposition of a Gaussian trough/hollow located at X = 0 mm and Y = −0.25 mm. The hollow shown on the left graph was defined as a 2D Gaussian with a Sigma of 2.4 mm and a central depth of 50 μm. This surface is intended to simulate corneal topography after myopic LASIK with refractive correction of about 3.5 dioptres and a slightly decentred optical zone displaced by 0.25 mm in the inferior direction. The surface is defined on a Cartesian grid within the central 8 mm zone with a lateral sampling of 100 points in both X and Y (in total 7668 data points). Dimensions for X, Y and Z data are in mm. (b) Fit error (FE) with a best fit floating spherical surface model (BFS, upper left graph), a best fit floating conic surface model (BFC, upper middle graph), a best fit floating biconic surface model (BFBC, upper right graph), a best fit floating spherical surface (as defined with BFS) with a fringe Zernike surface of radial degree N_Z_ = 12 on top (BFSZ, lower left graph), a fringe Zernike surface model of radial degree N_Z_ = 12 (BFZ, lower middle graph) and a custom Gaussian process surface model (BFGP, lower right graph) for surface C. The root‐mean‐squared fit error is shown in a millimetre scale and the root‐mean‐squared fit error (RMS) is provided in the title of the graph. Note that the colour maps are normalised in each case to the range of values in each plot.

**FIGURE 4:**
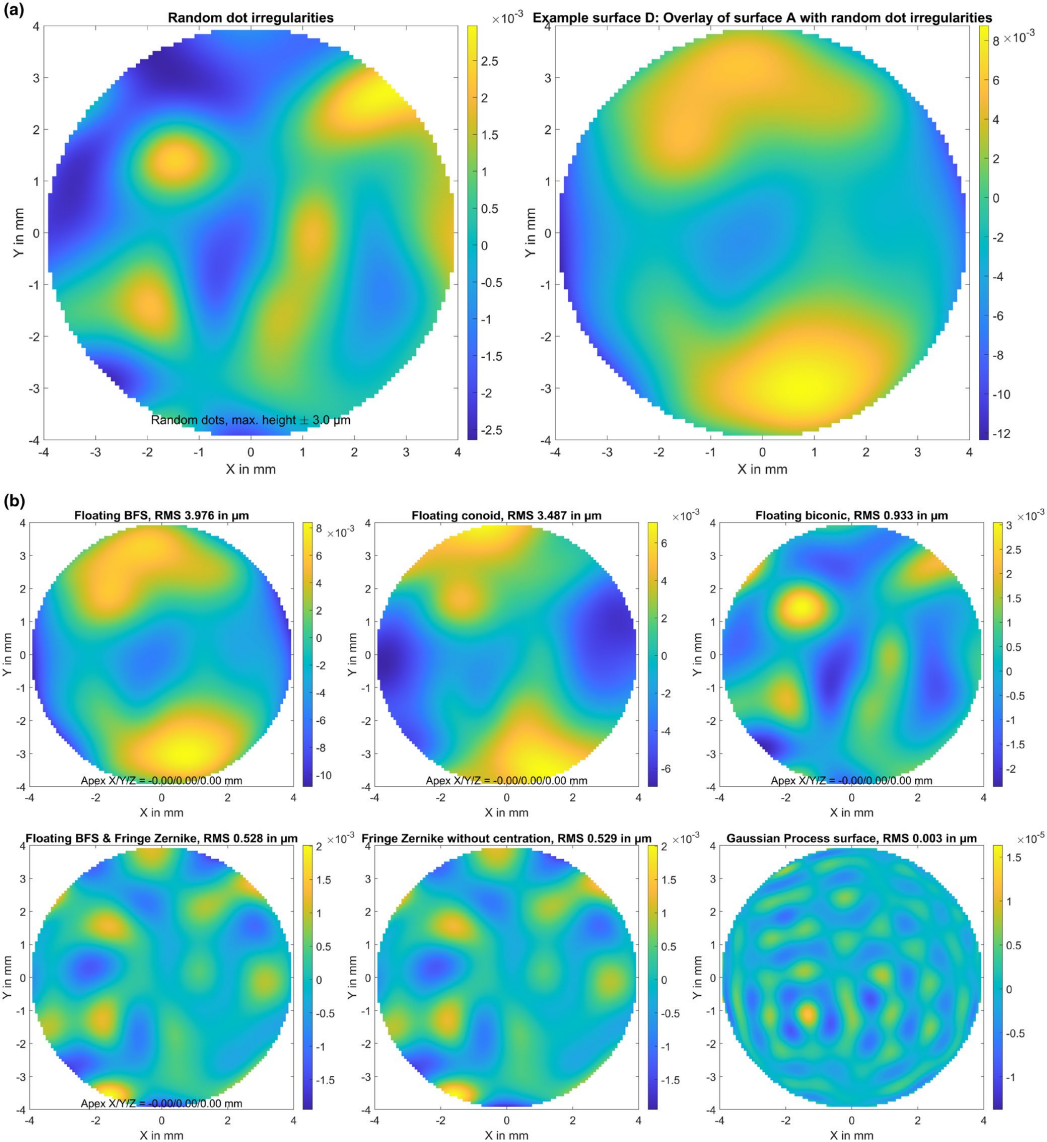
(a) Elevation maps of the generic surface D in terms of height difference from a best fit floating sphere. Surface D refers to the biconic surface from Figure 1a with a superposition of topographic irregularities randomly spread over the entire area of 8 × 8 mm. The height of the random dots varied between −3.0 and 3.0 μm, and a bicubic spline grid interpolation was used to interpolate between the random dots. This surface is intended to simulate a situation of corneal topographic irregularities. The surface is defined on a Cartesian grid within the central 8 mm zone with a lateral sampling of 100 points in both X and Y (in total 7668 data points). Dimensions for X, Y and Z data are in mm. (b) Fit error (FE) with a best fit floating spherical surface model (BFS, upper left graph), a best fit floating conic surface model (BFC, upper middle graph), a best fit floating biconic surface model (BFBC, upper right graph), a best fit floating spherical surface (as defined with BFS) with a fringe Zernike surface of radial degree N_Z_ = 12 on top (BFSZ, lower left graph), a fringe Zernike surface model of radial degree N_Z_ = 12 (BFZ, lower middle graph) and a custom Gaussian Process surface model (BFGP, lower right graph) for surface D. The root‐mean‐squared fit error is shown in a millimetre scale and the root‐mean‐squared fit error (RMS) is provided in the title of the graph. Note that the colour maps are normalised in each case to the range of values in each plot.

**FIGURE 5:**
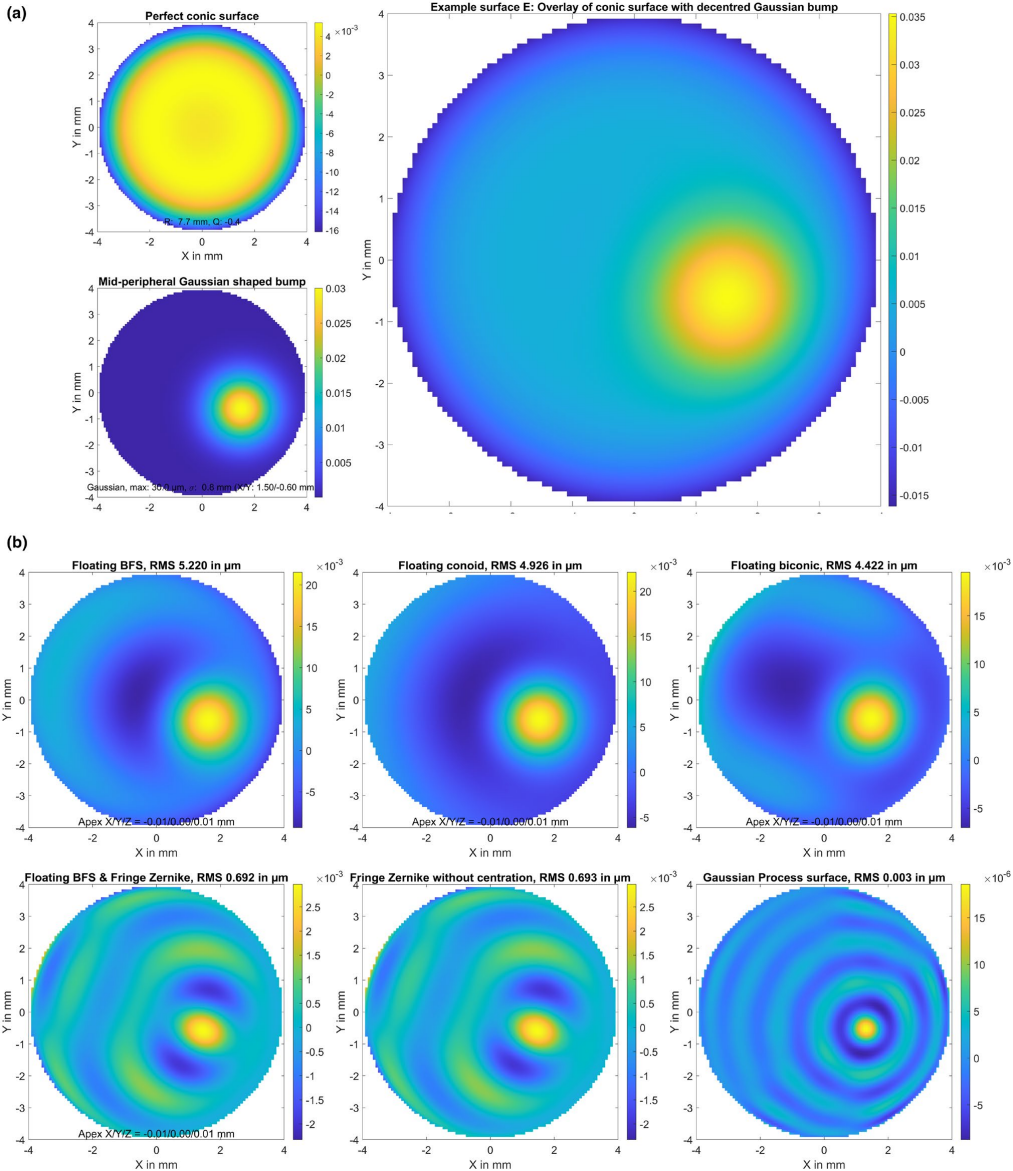
(a) Elevation maps of the generic surface E in terms of height difference from a best fit floating sphere. Surface E refers to a rotationally symmetric conic surface (upper left graph), with a Gaussian‐shaped bump superimposed in the mid‐periphery located at X = 1.5 mm and Y = −0.6 mm (sigma: 0.8 mm, maximum height: 30.0 μm, lower left graph). The resulting surface is shown on the right graph. This surface is intended to simulate a situation of corneal topography with keratoconus, with steepening in the lower temporal quadrant (left eye). The surface is defined on a Cartesian grid within the central 8 mm zone with a lateral sampling of 100 points in both X and Y (in total 7668 data points). Dimensions for X, Y and Z data are in mm. (b) Fit error (FE) with a best fit floating spherical surface model (BFS, upper left graph), a best fit floating conic surface model (BFC, upper middle graph), a best fit floating biconic surface model (BFBC, upper right graph), a best fit floating spherical surface (as defined with BFS) with a fringe Zernike surface of radial degree N_Z_ = 12 on top (BFSZ, lower left graph), a fringe Zernike surface model of radial degree N_Z_ = 12 (BFZ, lower middle graph) and a custom Gaussian process surface model (BFGP, lower right graph) for surface E. The root‐mean‐squared fit error is shown in a millimetre scale and the root‐mean‐squared fit error (RMS) is provided in the title of the graph. Note that the colour maps are normalised in each case to the range of values in each plot.

In this simulation study, a number of generic surface height data sets were generated and used to simulate corneal topography data. All of these generic surfaces were represented as a Cartesian grid with a lateral sampling in X and Y of 100 × 100 = 10,000 equidistant points within an area of 8 × 8 mm and a height Z. Data points with a distance (X^2^ + Y^2^) of more than 4 mm were discarded, leaving a circular region having a radius of 4 mm. The following generic surface data sets were created:

- A biconic surface (A) with radius of 7.8/7.6 mm and asphericity of −0.2/−0.4 in the flat/steep meridians, respectively. The flat meridian was oriented at 15 degrees. The surface elevation map in terms of the height difference Z minus a best fit floating sphere is displayed in Figure 1a, together with the parameters of the best fit sphere (apex position in X/Y/Z and radius of curvature R).
- The biconic surface defined in B, with decentration of 0.1 mm in X, 0.2 mm in Y and a superposition of random white noise in Z with a standard deviation of 2 μm (surface B). The random white noise and the resulting elevation map of this generic surface are shown in Figure 2a.
- The biconic surface defined in A, with superposition of a Gaussian trough/hollow at X = 0 mm and Y = −0.25 mm. The hollow was defined as a 2D Gaussian with a sigma of 2.4 mm and a central depth of 50 μm (surface C). The hollow and the resulting elevation map of the model surface are shown in Figure 3a. This generic surface is intended to simulate corneal topography after myopic LASIK with refractive correction of about 3.5 dioptres and a slightly decentred optical zone displaced by 0.25 mm in the inferior direction.
- The biconic surface defined in A, with superposition of some topographic irregularities randomly spread over the entire area of 8 × 8 mm. The height of the random dots varied between −3.0 and 3.0 μm and a bicubic spline grid interpolation was used to smooth between the random dots (surface D). The topographic irregularities and the resulting elevation map of the model surface are displayed in Figure 4a. This generic surface is intended to simulate corneal topographic irregularities.
- A rotationally symmetric conic surface with radius R = 7.7 mm and asphericity Q = −0.4, with a superimposed bump in the mid‐periphery located at X = 1.5 mm and Y = −0.6 mm. The bump consists of a 2D Gaussian with a sigma of 0.8 mm and a maximum height of 30.0 μm (surface E). The rotationally symmetric conic surface (upper left graph), the mid‐peripheral bump (lower left graph) and the resulting elevation map of the model surface (right graph) are shown in Figure 5a. This generic surface is intended to simulate corneal topography with a keratoconic eye, with steepening in the lower temporal quadrant (left eye).

The generation of the generic surfaces and the subsequent surface fit using various surface models was implemented in MATLAB (Matlab version 2022b, mathworks.com). The following surface models were fitted to the generic surfaces described above (A–E):

BFS: A floating best fit sphere with three degrees of freedom (apex offset in X/Y/Z and radius R^8^).

BFC: A floating best fit rotationally symmetric conoid with four degrees of freedom (apex offset in X/Y/Z and radius R^8^).

BFBC: A floating best fit biconic surface with eight degrees of freedom (apex offset in X/Y/Z, radii in the flat and steep meridian R1 and R2, respectively, asphericity in the flat and steep meridian Q1 and Q2, respectively, and orientation of the flat axis A1).^8^

BFSZ: A floating best fit sphere (as described in BFS) with a superimposed fringe Zernike polynomial surface of radial degree N_Z_ = 12 (as described in detail in Langenbucher et al.^11^) with a reference diameter of 4 mm (= unit circle), having four degrees of freedom for the BFS fit and 36 Zernike polynomials for the fringe Zernike fit (Z1 to Z36).

BFZ: A best fringe Zernike polynomial surface of radial degree N_Z_ = 12 (as described in detail in Langenbucher et al.^11^) with a reference diameter of 4 mm (= unit circle) without centring, with 36 Zernike polynomials for the fringe Zernike fit (Z1–Z36).

BFGP: Best fit Gaussian process surface with adaptive sigma and kernel size. A squared exponential design was used for the covariance function. The parameters of the Gaussian process model were estimated on a random subset of 2000 data points. A Quasi‐Newton algorithm was used for optimisation.^19^ Since the BFGP surface is fully customised without symmetry conditions, there was no need to extract the apex position.^7^, ^13^, ^14^, ^15^, ^16^, ^17^, ^20^, ^21^, ^22^

An iterative nonlinear optimisation technique (trust‐region‐reflective^23^, ^24^) was used to extract the parameters for all models except the BFGP. The selected optimisation metric was minimisation of the root‐mean‐squared model error, defined as the unweighted difference between the surface data (surfaces A–E) and the fitted model data at the 7668 data points within a radius of X^2^ + Y^2^ ≤ 4 mm.

### Output data and quality metrics

For all five generic surfaces and six surface‐fitted models, the surface parameters were documented for BFS, BFC, BFBC, BFSZ, BFZ and the fit error (FE), defined as the difference between the surface height Z and the height of the fitted model surfaces, together with the unweighted root‐mean‐squared (RMS) value of the FE.^19^, ^23^, ^24^, ^25^, ^26^

## RESULTS

The characteristics of the six surface models, as fitted to each of the generic surfaces A–E, are listed in Table 1 together with the corresponding root‐mean‐square fit error (RMS FE).

**TABLE 1:** Fit results for the various surface models as fitted to the generic surfaces (A)–(E).

Surface fit		Surface A	Surface B	Surface C	Surface D	Surface E
BFS	Apex position X/Y/Z	0.000/0.000/0.004	0.098/0.299/0.004	0.000/−0.005/−0.029	−0.002/0.001/0.004	−0.008/0.003/0.012
	R in mm	7.862	7.868	7.624	7.867	7.969
	RMS FE in μm	4.114	4.622	8.231	3.976	5.220
BFC	Apex position X/Y/Z	0.000/0.000/0.000	0.099/0.302/0.000	0.000/−0.006/−0.045	−0.002/0.001/0.000	−0.008/0.003/0.008
	R in mm/Q	7.699/−0.302	7.702/−0.297	7.021	7.696/−0.317	7.810/−0.300
	RMS FE in μm	3.685	4.134	4.131	3.487	4.926
BFBC	Apex position X/Y/Z	0.000/0.000/0.000	0.100/0.300/0.000	0.000/−0.006/−0.045	−0.002/0.001/0.000	−0.008/0.003/0.008
	R1 in mm/Q1/A1 in°	7.800/−0.200/15.0	7.796/−0.211/15.0	7.105/−0.9991/15.4	7.793/−0.211/12.6	7.970/0.004/68.2
	R2 in mm/Q2	7.600/−0.400	7.6001/−0.4001	6.9399/−1.1301	7.6001–0.4196	7.6660–0.6058
	RMS FE in μm	8.4958 10^−9^	1.995	2.032	0.933	4.422
BFSZ	Z2/Z3 in μm	0.0000/0.000	0.000/0.000	0.000/0.000	0.000/0.001	0.000/0.000
	Z4/Z5/Z6 in μm	−0.000/−0.001/0.002	−0.000/−0.001 0.002	−0.000 −0.001 0.002	−0.000/−0.001/0.002	−0.005/−0.001/−0.001
	Z9 in μm	−0.001	−0.001	−0.004	−0.001	−0.003
	RMS FE in μm	1.68 10^−4^	1.988	0.008	0.528	0.692
BFZ	Z2/Z3 in μm	0.000/0.000	−0.014/−0.042	0.000/0.001	0.000/−0.000	0.001–0.000
	Z4/Z5/Z6 in μm	0.137/−0.001/0.002	0.136/−0.001/0.002	0.141/−0.001/0.002	0.136/−0.001/0.002	0.130/−0.001/−0.001
	Z9 in μm	0.002	−0.001	−0.000	0.002	0.001
	RMS FE in μm	1.66 10^−4^	1.993	0.008	0.529	0.693
BFGP	RMS FE in μm	2.49 10^−4^	1.995	8.90 10^−4^	0.003	0.003

*Note*: For the BFSZ and BFZ models, from the 36 fringe Zernike coefficients, only the two tilt components Z2 and Z3, the defocus and astigmatism components Z4, Z5 and Z6 and the spherical aberration component Z9 are listed. RMS FE refers to the root‐mean‐squared fit error of the surface models for generic surfaces (A)–(E), respectively. Apex position refers to the X, Y and Z coordinates of the highest surface point, R to the overall surface radius, R1 and R2 to the radii in the flat and steep meridians, Q to the overall surface asphericity, Q1 and Q2 to the surface asphericity in the flat and steep meridians.

Abbreviations: BFBC, floating best fit biconic surface; BFC, floating best fit rotationally symmetric conoid; BFGP, custom Gaussian process model surface; BFS, floating best fit sphere; BFSZ, best fit floating sphere (respective apex position and R as shown for BFS) and a fringe Zernike surface with radial degree N_Z_ = 12 on top; BFZ, fringe Zernike surface fit with radial degree N_Z_ = 12.

Figure 1b shows the FE derived with various surface models as fitted to the perfect biconic generic surface (surface A). The FE for the BFS surface in the upper left graph shows that the fitted surface model was perfectly centred, but cannot represent the astigmatism and the asphericity of the generic surface A. The FE for the BFC indicated that the fitted surface model was able to follow the overall asphericity of surface A, but not in both cardinal meridians. Furthermore, it cannot represent the astigmatism. The FE for the BFBC surface shows the best fit of all of the surface models, giving a good fit to the radius of curvature and asphericity in both cardinal meridians, as well as the orientation of the flat meridian of curvature as indicated by FEs of the order of 10^−11^ mm. Even though the BFBC graph visually resembles the surface elevation shown in Figure 1a, suggesting some correlation between the elevation and FE, the scales are very different: at 8.5 10^−9^, the RMS FE was very small, close to the numerical precision of the fitted algorithm. The FE for the BFSZ with an underlying BFS and for the BFZ surface model, as shown in the lower left and lower middle graphs, are quite similar. Both surface models provide good fits to the astigmatism and the asphericity of surface A, with FEs of the order of 10^−7^ mm. These two plots show patterning in the same orientation as the variations in the original surface elevation, although these should again be taken in the context of the much smaller scale. At 10^−6^ mm in the centre, the FE for the BFGP surface model fit error was slightly worse than the FE with BFSZ and BFZ, although it appears azimuthally symmetric, with no correlation to the original surface elevation.

Figure 2b displays the FE derived with various surface models as fitted to the perfect biconic surface A with decentration and superposition of white noise (surface B). The FE graphs for the BFS, BFC and BFBC indicate that all three fitted surface models were able to represent fully the surface decentration (apex position), even with noisy data. The model characteristics listed in Table 1, such as radii and asphericity (compared with the perfect biconic surface A) are also well represented by these models. The surface models BFBC, BFSZ, BFZ and BFGP, which are, in principle, suitable to represent biconic surfaces, provide an RMS FE which matches the standard deviation of the white noise, as shown in Figure 2a. The Zernike model BFZ provides no option for dealing with the decentration of a surface and interpreted the surface decentration in X and Y mostly in terms of surface tilt as shown in Table 1. Surprisingly, this model showed similar performance to the BFSZ surface model, where the surface decentration was covered by the underlying BFS surface.

Figure 3b plots the FE derived with various surface models as compared with the perfect biconic surface A, with superposition of a Gaussian shaped hollow slightly decentred in the inferior direction (surface C). The graphs show that the BFS model surface (upper left graph) exhibits very poor performance, with an RMS FE >8 μm, followed by the BFC (upper middle graph) at 4.1 μm and the BFBC (upper right graph) at 2.0 μm. In contrast, the BFSZ and BFZ Zernike surface models show good performance, each having an RMS FE around 0.008 μm. The BFGP model surface performed best with an RMS FE of about 0.001 μm. Interestingly, both of the Zernike representations and the Gaussian Process representation display some radial oscillations in the FE profile.

Figure 4b shows the FE derived with various surface models as fitted to the perfect biconic surface A, with superposition of multiple random dots representing topographic surface irregularities (surface D). Again, the graphs show that the BFS surface model (upper left graph) exhibits very poor performance, with an RMS FE of about 4 μm, followed by the BFC (upper middle graph) at 3.5 μm and the BFBC (upper right graph) at 0.9 μm. In the FE plot for the BFS and BFC, the basic surface elevation is still visible, but in the FE plot for the BFBC, the biconic configuration is almost recovered and the random dots are dominant. Interestingly, both Zernike model surfaces BFSZ (lower left graph) and BFZ (lower middle graph) mostly recover the biconic shape, but cannot follow the surface irregularity in terms of the random dots. As a result, the RMS FE for both Zernike surface models is about 0.5 μm, which is significantly poorer than their performance on the first three surfaces. In contrast, the Gaussian process surface model BFGP (lower right graph) shows a very good performance in modelling local irregularities, with an RMS FE in the range of 0.003 μm.

The graphs shown in Figure 5b are based on a rotationally symmetric centred conoid surface with superposition of a Gaussian shaped bump in the mid‐periphery representing a ‘corneal ectasia’ (surface E). Again, the BFS surface model in the upper left graph exhibits very poor performance, producing an RMS FE of 5.2 μm. This is followed by the BFC (upper middle graph) at 4.9 μm and the BFBC (upper right graph) at 4.4 μm. The BFS, BFC and BFBC surface models appear unsuitable to represent such a bump in the mid‐periphery. However, even the two Zernike representations BFSZ and BFZ (lower left and middle graphs) cannot model such an ectatic surface configuration very closely, yielding an RMS FE of about 0.7 μm. In contrast, the BFGP surface performed surprisingly well, with an RMS FE of about 0.003 μm, even though the bump is still visible in the FE graph.

## DISCUSSION

The majority of topographers or tomographers generate map data in terms of surface curvature, elevation or height. Surfaces are typically represented with thousands of measurement points organised in a Cartesian or cylindrical grid. In general, the measurement data could also be organised as a scattered grid. While these are purely mathematical descriptions, in practice one would normally wish to assess the cornea for clinically relevant effects such as asphericity or toricity, or for nonuniformity associated with ectasia (for example). For these applications, a continuous surface model fitted to the data grid can be useful. In the context of the current study, one may also wish to use ray tracing to assess image quality. However, such data grids are not sufficient for applications such as ray tracing, as information about the surface and the normal to the surface in the interspaces between the data points is not available. In general, a proper surface model is fundamental for all ray tracing applications when working with point cloud data.^3^, ^13^, ^14^ Regardless of the accuracy of the measured data points, there is no information as to the appearance of the surface between the sampling points.^4^ Making the real‐world assumption that the data are subject to a certain level of noise, it is typically necessary to use approximation strategies to counterbalance the FE and surface smoothness. Spherical or second‐order surfaces such as conoids^1^, ^4^, ^8^ have the advantage that the ray surface intersection and the surface normal can easily be derived algebraically, reducing the mathematical complexity of ray tracing. However, more complex surfaces which offer more flexibility in following surface configurations with local irregularities require iterative nonlinear strategies to derive the ray‐surface intersections. In these cases, calculation of the normal to the surface could be achieved by applying numerical methods such as the vector product of the two surface gradients with respect to X and Y.^2^, ^7^, ^10^, ^11^

Surface models fitted to the data grid could also be used for research into customised intraocular lenses. For example, the complete imaging path of the eye could be ray traced, starting with a customised corneal model in which both corneal surfaces are characterised in terms of a BFGP, BFSZ or BFZ fitted surface. This would be followed by an aperture stop (representing the pupil) and a standard intraocular lens (either spherical or aspheric). Ray tracing could then be used to evaluate the resulting wavefront error in terms of optical path length differences. It would be possible to iteratively refine one (or both) intraocular lens surfaces using a Gaussian process surface model, reducing the wavefront error and predicting the resulting retinal image performance. This iteration process would be continued until a satisfactory image quality is obtained.

Other applications of surface modelling include the generation of corneal ablation profiles for excimer laser treatment. Current corneal tomographers consider the cornea either as a monolayer with two surfaces (front and back surface) or as a duolayer with three surfaces (front, back and the epithelium–stroma interface). These two‐ or three‐surface models could be used to generate customised ablation profiles characterised in terms of Gaussian process models iteratively enhanced to optimise the imaging performance of the cornea. In the present study, five characteristic generic corneal surface data sets were used to assess the performance of various surface fitting representations. The representations evaluated included classical BFS, BFC and BFBC models, as well as more advanced models such as fringe Zernike polynomials^10^, ^11^, ^12^ and a Gaussian Process surface commonly used in machine learning applications.^9^, ^13^, ^14^, ^15^, ^16^, ^17^, ^18^, ^20^, ^21^, ^22^, ^27^, ^28^ The results for surface A are not surprising. Since the biconic surface has eight degrees of freedom, the BFS model (with only four degrees of freedom) was not capable of retrieving the astigmatism and asphericity. The BFC surface was capable of retrieving the mean asphericity but not the astigmatism. However, for clinical applications with ray tracing, the BFBC, BFSZ, BFZ and BFGP models performed quite well.

It was surprising that decentration of this biconic surface (B) and the superposition of white noise did not affect the performance of the models significantly. The BFS, BFC and BFBC models were all capable of retrieving the surface decentration fully, as well as the surface parameters such as radius and asphericity, which seemed to be unaffected by the decentration and white noise.

The third configuration (C) modified the biconic surface by subtracting a Gaussian shaped profile paracentrally to simulate the situation after myopic LASIK with a central ablation of 50 μm. The classical surface models BFS, BFC and BFBC appear overwhelmed in such situations, resulting in a large FE. However, even though the Zernike representations BFSZ and BFZ and the Gaussian Process representation BFGP performed much better, they still could not follow the paracentral hollow fully. Here, the BFGP model showed the best performance. Although the Zernike models appeared unaffected by small levels of decentration, they did so by interpreting decentration in terms of tilt. Since the BFGP model can account for decentration directly, it is expected that the superiority of the BFGP model in this respect would become more apparent at larger decentrations.

The greater flexibility of the BFGP model was also seen clearly in surface D, where random dots were superimposed onto the biconic surface to test the robustness of the surface fit to local corneal irregularities. Again, it is not surprising that the classical surface models BFS, BFC or BFBC did not perform well, being able to model the basic shape only, but not the irregularities. However, it was somewhat surprising that the fringe Zernike models of radial degree N_Z_ = 12, either on top of a BFS (BFSZ) or with a direct decomposition (BFZ) also did not perform well. This means that Zernike surfaces are not suitable models for ray tracing in cases with local irregularities. For both Zernike models, the FE ranged between −2 and +2 μm, and the RMS FE was 0.5 μm even with the random dots of maximum height of ±3 μm. In contrast, the BFGP model seems to be more flexible in adapting to local irregularities, especially with the selection of a suitable Kernel function and size. Although some surface roughness is still visible in the FE profile, the RMS FE was very low at only 0.003 μm with this surface model.

However, the largest challenge for these surface models was the situation of a very simple centred conoid surface with a Gaussian‐shaped bump superimposed in the mid periphery (generic surface example E). This was used to simulate the situation of a corneal ectasia typically located in the lower temporal quadrant. Again, it is not surprising that classical surface models such as the BFS, BFC or BFBC were overwhelmed by such topographic situations. However, it was rather surprising that the Zernike models also struggled. Even with the 36 Zernike polynomial terms, either on top of a BFS (BFSZ) or fitted directly to the surface height data of surface (E), the residual FE was in the range of 0.7 μm, which is disappointing. The bump is clearly visible in the FE graphs with BFSZ and BFZ and surrounded by some rings of waviness. Furthermore, even the BFGP surface model could not fully retrieve surface (E) and the bump is also clearly visible in this example. However, the FE with the BFGP surface model was in the range of ±0.02 μm (RMS FE = 0.003 μm) which was quite good.

It was surprising to not find any clinically relevant difference between the two Zernike representations. Since Zernike polynomials all follow some symmetry, it might be anticipated that the subtraction of a floating BFS from the height data before performing the Zernike fit might be a good option for coping with surface decentration (e.g., surface B). However, it was actually found that the Zernike fit interpreted decentration in terms of either tilt (as shown in Table 1) or coma (not listed in the Table), and the overall performance of the fit seemed to be unaffected, at least for small values of decentration.^11^

In this respect and with regard to surface irregularities, the BFGP model showed the greatest flexibility because the wavelets were simply shifted and not subject to any symmetry conditions. BFGP accounts for decentration intrinsically and was able to cope with local isolated defects.

However, this study has some limitations: First, the analysis was restricted to some (characteristic, but selected) generic test surfaces and selected surface models for the fit. This does not allow for general statements about the robustness of any surface model in characterising individual corneal surface data. The literature shows that classical BFS, BFC, BFBC or Zernike surfaces, as evaluated here, have been used widely in the past to characterise topographic data. However, there are many other surface models which were not considered here. Second, the study was restricted to fitting the test surfaces with model surfaces. In subsequent papers, we plan to investigate how these simplified surface models impact image quality using ray tracing. This further analysis, in terms of image quality, was not pursued here to avoid confusing several effects. And third, the trust region reflective algorithm^23^, ^24^ was used as the nonlinear iterative optimisation technique (for BFS, BFC, BFBC, BFSZ and BFZ) and the quasi‐Newton algorithm for optimisation^19^ of the BFGP. It was felt that other iterative nonlinear optimisation strategies such as the Levenberg–Marquardt algorithm or the interior point algorithm would show similar performance, and that all iterative nonlinear algorithms^24^, ^25^, ^26^ may outperform the classical least squares set‐up often adopted to derive Zernike polynomials using the Penrose–Moore pseudoinverse, although there is no proof for this assumption.

In conclusion, this paper addresses the problem of defining a closed surface model from point cloud data derived from surface topographers or tomographers used for ray tracing. Since surface maps from tomographers are typically provided as grid data, one must find a trade‐off between noise and the reliability of the data points, and this was achieved through approximation of a surface model to the point cloud data. From the results with characteristic test surfaces, it was found that classical surfaces such as best fit spheres or best fit conoids might be a satisfactory and easy‐to‐use option for very smooth topographies, that is, best fit biconic surfaces could best represent smooth topographies with astigmatism and asphericity. However, Zernike surfaces with or without an underlying best fit sphere offer much more flexibility, for example, to represent situations after corneal refractive surgery. Finally, Gaussian process surfaces show surprisingly good performance and greater flexibility in coping with decentration and adapting to local irregularities or ectatic corneas, making these a compelling all‐round option. Future studies will investigate the Gaussian process surface model for characterising surfaces of customised intraocular lenses and applications in the field of custom surface ablations with the excimer laser.

## AUTHOR CONTRIBUTIONS

**Achim Langenbucher:** Conceptualization (equal); formal analysis (equal); investigation (lead); methodology (lead); writing – original draft (lead). **Nóra Szentmáry:** Project administration (equal); supervision (equal); writing – original draft (supporting). **Alan Cayless:** Methodology (supporting); visualization (equal); writing – original draft (equal). **Peter Hoffmann:** Conceptualization (equal); data curation (equal); project administration (equal); visualization (supporting). **Jascha Wendelstein:** Formal analysis (equal); resources (supporting); software (equal); validation (equal).

## FUNDING INFORMATION

None.

## CONFLICT OF INTEREST STATEMENT

The authors report no conflicts of interest and have no proprietary interest in any of the materials mentioned in this article.
